# Bilateral Matching Method for Business Resources Based on Synergy Effects and Incomplete Data

**DOI:** 10.3390/e26080669

**Published:** 2024-08-06

**Authors:** Shuhai Wang, Linfu Sun, Yang Yu

**Affiliations:** 1 School of Computing and Artificial Intelligence, Southwest Jiaotong University, Chengdu 611756, China; 2 Manufacturing Industry Chain Collaboration and Information Support Technology Key Laboratory of Sichuan Province, Southwest Jiaotong University, Chengdu 610031, China; 3 School of Computer Science and Software Engineering, Southwest Petroleum University, Chengdu 610500, China

**Keywords:** bilateral matching, synergy effect, business resources, fuzzy analytic hierarchy process, entropy weight method, data analytics

## Abstract

On the third-party cloud platform, to help enterprises accurately obtain high-quality and valuable business resources from the massive information resources, a bilateral matching method for business resources, based on synergy effects and incomplete data, is proposed. The method first utilizes a k-nearest neighbor imputation algorithm, based on comprehensive similarity, to fill in missing values. Then, it constructs a satisfaction evaluation index system for business resource suppliers and demanders, and the weights of the satisfaction evaluation indices are determined, based on the fuzzy analytic hierarchy process (FAHP) and the entropy weighting method (EWM). On this basis, a bilateral matching model is constructed with the objectives of maximizing the satisfaction of both the supplier and the demander, as well as achieving the synergy effect. Finally, the model is solved using the linear weighting method to obtain the most satisfactory business resources for both supply and demand. The effectiveness of the method is verified through a practical application and comparative experiments.

## 1. Introduction

The third-party cloud platform provides support for business collaboration for various enterprises, such as suppliers, distributors, service providers, 4S shops, and logistics providers. As the number of enterprises on the third-party cloud platform grows, so does their business collaboration, resulting in the accumulation of a large number of business resources [1,2]. These business resources include data resources, process resources, service resources, product resources, etc., which can help enterprises improve supply chain management efficiency, strengthen business collaboration, perform data analysis, provide decision support, and more. These bring greater competitive advantages and development opportunities to enterprises. However, with the continuous increase of business resources, it is difficult for enterprise users on the cloud platform to obtain high-quality and valuable resources that meet their own requirements. This leads to the problem of information overload. Therefore, on the third-party cloud platform, how to quickly and accurately obtain high-quality and valuable business resources from complex and massive information resources is one of the key problems in improving an enterprise’s competitiveness and operational efficiency. To meet this challenge, we adopt a bilateral matching method. The method integrates the personalized business needs of both the supply and demand sides, selecting the most satisfying business resources for both parties from a vast array of information resources [2]. By optimizing the matching relationship between the supply and demand sides, it helps enterprises to efficiently identify the required resources, reduce information redundancy, and achieve an overall optimal collaboration effect between suppliers and demanders. In the field of cloud manufacturing, many scholars have conducted extensive studies on the matching problem, focusing on aspects such as research and development (R&D) tasks, quality of service (QoS), and business resources [1,2].

In terms of R&D tasks, Lu et al. [3] proposed a truthful double auction mechanism, to address the problem of matching users’ task requirements and providers’ resources in bilateral cloud markets. This mechanism uses Lyapunov optimization technology to minimize the cost for users, which is beneficial for both the cloud service provider and the user. Li et al. [4] proposed a novel two-sided matching model based on dual hesitant fuzzy preference information, to solve the fuzziness and uncertainty of preference information in the matching process of complex product manufacturing tasks on the cloud manufacturing platform. Liu et al. [5] proposed a task assignment method based on bilateral matching (TAMBM) between subtask and designer, to address the problem of collaborative design subtasks assignment in design teams. They constructed a multi-objective optimization model for collaborative design task allocation based on bilateral matching, and they used the improved sparrow search algorithm to solve the model. In terms of QoS, Hao et al. [6] proposed a QoS-based two-sided matching model of cloud services, in order to solve the problem of on-requirement mutual selection of service providers and tasks in a cloud manufacturing environment. For the manufacturer–dealer bilateral adaptation problem in the intelligent cloud manufacturing environment, Fang et al. [7] proposed a new bilateral adaptation algorithm based on Q-learning and an improved Gale–Shapley algorithm, to gain superior results. In order to evaluate and optimize the adaptability of service-matching strategies, Xue et al. [8] proposed a computational experiment-based evaluation framework for service-matching strategies, which can simulate all kinds of actual scenarios, to verify the performances of service-matching strategies. In terms of business resources, Yu et al. [2] proposed a business resource bilateral matching model (BRBMM), which can accurately obtain high-quality and valuable business resources from massive information resources. In order to improve the accuracy of matching decisions between manufacturing service resources and tasks in a cloud environment, Xiao et al. [9] proposed a matching decision method for manufacturing service resources, which considers multiple influencing factors in resource matching. In other applications, Wang et al. [10] proposed a two-sided matching model (TMM), to address the challenges of information asymmetry and low matching efficiency in the freight market by leveraging adverse user behaviors to enhance platform matching efficacy. To solve personnel–position matching issues, Yu et al. [11] introduced an intuitionistic fuzzy two-sided matching model (IFTMM), which employs novel intuitionistic fuzzy Choquet integral aggregation operators to describe correlations between evaluation attributes, and which effectively enhances accuracy in personnel–position matching.

The above methods studied the matching problem from different angles and achieved good results. However, they relied on matching the complete data. When the cloud platform cleans and organizes the received data, issues such as poor data quality and inconsistent data formats may lead to data loss [12]. This will impact the accuracy of the matching. Also, existing matching methods based on business resources rarely consider synergy effects. Therefore, the above methods cannot be perfectly applied to business resource matching on the third-party cloud platform, and there is a common problem of poor accuracy in the matching process. To solve these problems, this paper proposes the bilateral matching-method for business resources (BMBR) based on synergy effects and incomplete data. In order to solve the problem of missing data, the method firstly uses the comprehensive similarity-based k-nearest neighbor imputation algorithm (CSKI) to fill in the missing values. Then, a satisfaction evaluation index system is constructed, and the weights of the satisfaction evaluation indexes are determined based on the FAHP and the EWM. On this basis, a two-sided matching model is established based on synergy effects. Finally, the linear weighting method is applied to solving the model, in order to obtain the most satisfactory business resources for both the supply and demand sides.

The main contributions of this study are as follows: (1) Proposing CSKI for filling in missing values, which combines a business resource attributes-based similarity measure and a hybrid difference-based similarity measure. (2) Constructing a satisfaction evaluation index system based on the demander’s requirements and the supplier’s preferences. (3) The synergy effect is is determined by the the collaboration requirements between business resource demanders and suppliers. The two-sided matching model is established based on the synergy effects. (4) We conducted experiments on six different business resource datasets, and the results demonstrate that our proposed method can effectively improve matching accuracy compared to the state-of-the-art matching methods, and that it can enhance overall satisfaction for both parties.

The remaining sections of this paper are organized as follows: Section 2 introduces the proposed method. In Section 3, we present an example application and comparative analysis of this article. The paper is concluded in Section 4.

## 2. Methodology

**Figure 1:**
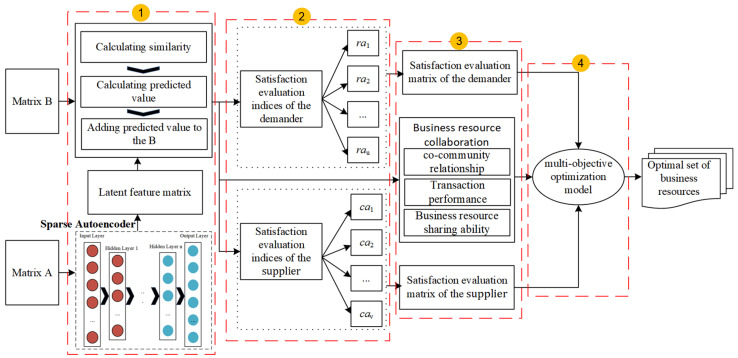
The structure of the proposed method.

The main structure of the proposed BMBR method is illustrated in Figure 1. It contains four components: (1) imputation of missing values; (2) constructing a satisfaction evaluation index system; (3) normalization of the satisfaction evaluation index values; (4) construction and solution of a multi-objective optimization model. In Figure 1, *B* is is a matrix formed by randomly extracting evaluation index-related data resources from the data space of the third-party cloud platform, and *A* is a matrix formed by extracting the attribute information of business resources from the data space of the third-party cloud platform.

### 2.1. Imputation of Missing Values

This section introduces CSKI, which aims to fill in missing values in *B* for accurate matching. The principle of CSKI is to find the $k^{\prime }$ data points that are most similar to the missing data based on the existing data points, and then, to use them to predict and fill in the missing values. To calculate the missing values, we need to evaluate the similarity values between each pair of business resources.

#### 2.1.1. Quantification of the Textual Data

The original business resource dataset comprises text data that require conversion into numerical form for bilateral matching. To facilitate this conversion, a text convolutional neural network (TextCNN) is employed. Subsequently, the text data are classified into five categories: excellent (5 points), good (4 points), average (3 points), poor (2 points), and very poor (1 point).

TextCNN is a text classification model based on convolutional neural networks (CNN). It usually consists of the following four layers: input layer, convolution layer, pooling layer, and fully connected layer. The input layer converts *B* into a matrix of word embeddings with dimensions n×k through the word2vec model, where *n* represents the number of words in the textual data and *k* represents the dimension of the word embedding matrix. The convolutional layer is used to extract local features. In this layer, convolutional kernels of sizes 2, 3, and 4 are employed, to capture relationships between different character spans. The pooling layer extracts important information from the feature maps computed by the convolutional layer [13]. In this layer, we utilize 1-max pooling for all convolutional kernels and then cascade them, to obtain the final feature vector. The fully connected layer serves as the last layer in the TextCNN model construction. It is built based on the output of the pooling layer and the number of classification categories. The softmax function is employed to obtain the ultimate classification results. In this layer, dropout is used to avoid overfitting.

#### 2.1.2. Business Resource Attributes-Based Similarity Measure

The similarity measure based on the business resource attributes is calculated by assessing the attribute information of the resources (e.g., type, quantity, volume, customer ID, etc.), to determine the similarity between business resources. This is called (SMBRA). The attribute information usually has large dimensions, which increases the computational complexity of the similarity calculation [14,15]. In this paper, we use a sparse autoencoder to reduce the dimensionality. The sparse autoencoder is a neural network model for unsupervised learning that can learn a set of meaningful feature representations from input data. It encodes the input data into a low-dimensional sparse representation by training a neural network with multiple hidden layers, and it then reconstructs the original input data, using a decoder. Compared to traditional autoencoders, sparse autoencoders incorporate sparsity constraints on the activation function of the hidden layers.

A sparse autoencoder consists of an encoder and a decoder. Suppose $A=\left\{X_{1},X_{2},\cdots \right.$$\left.,X_{i},\cdots ,X_{n}\right\}$ denotes the business resource attribute data; *n* denotes the number of training samples; $X_{i}=\left(x_{i1},x_{i2},\ldots ,x_{ip}\right)$ is a *p*-dimensional attribute vector. The encoder of the sparse autoencoder can be applied, to obtain the nonlinear representations of the input vectors [16]. The formulation of the encoder is shown as follows:

(1) h=f(WA+b)

where *h* is the feature vector, *W* denotes the weight matrix for the encoder, *f* represents the activation function, and *b* represents the bias vector for the encoder. The formulation of the decoder is shown as follows:

(2) $$Y=f^{\prime }\left(W^{\prime }h+b^{\prime }\right)$$

where $W^{\prime }$ represents the weight matrix for the decoder, $f^{\prime }$ is the activation function, $b^{\prime }$ denotes the bias vector for the decoder, and *Y* is the reconstruction of *A*. The objective of $F_{\cos t\;}$ is to minimize the reconstruction error between input and output:

(3) $$F_{\mathrm{cost}}=\frac{1}{n}\sum_{i=1}^{n}{∥Y_{i}-X_{i}∥}^{2}$$

We add an additional sparse penalty term, to optimize the objective function. The sparse penalty term $J_{\mathrm{sparse}\;}\left(\rho \right)$ is shown as follows:

(4) $$J_{\mathrm{sparse}\;}\left(\rho \right)=\sum_{m=1}^{q}\left(\rho \log\frac{\rho }{\rho_{m}}+\left(1-\rho \right)\log\frac{1-\rho }{1-\rho_{m}}\right)$$

(5) $$\rho_{m}=\frac{1}{n}\sum_{i=1}^{n}f\left(W_{m}X_{i}+b_{m}\right)$$

where $\rho_{m}$ denotes the average activation of the hidden unit m;ρ is the sparse parameter; and *q* is the number of hidden-layer neurons.

In addition, a regularization item that can penalize the weights of the network is added to the loss function, to avoid overfitting [16]. It is shown as follows:

(6) $$J_{\mathrm{weight}\;}\left(W\right)=\lambda_{1}\left({∥W∥}_{2}+{∥W^{\prime }∥}_{2}\right)$$

where $\lambda_{1}$ is the weight attenuation coefficient and $J_{\mathrm{weight}\;}\left(W\right)$ represents the sparse penalty term. Accordingly, the objective function of the sparse autoencoder is represented as follows:

(7) $$J_{\mathrm{loss}\;}\left(W,b\right)=F_{\mathrm{cost}\;}+J_{\mathrm{weight}\;}\left(W\right)+\mu J_{\mathrm{sparse}\;}\left(\rho \right)$$

where $J_{\mathrm{loss}\;}\left(W,b\right)$ is the overall objective loss function and μ is the weighting coefficient of the sparse penalty term.

The sparse autoencoder described above has just one hidden layer, so it has a limited ability to learn features from data. To improve its learning ability, it is important to build a deep sparse autoencoder that can effectively learn potential features from the business resource attribute data. Therefore, this paper utilizes the learning model proposed in the literature [17] to train a deep sparse autoencoder. In the training algorithm, the first hidden layer can be trained using the input data, and then the output obtained from the first hidden layer can be used to train the second hidden layer, and so on [18]. *A* is the input data of the deep sparse autoencoder. The latent features $X^{\prime }$ of *A* can be represented as follows:

(8) $$X^{\prime }=\begin{array}{c} X_{1}^{\prime }\\ \vdots \\ X_{i}^{\prime }\\ \vdots \\ X_{n}^{\prime } \end{array}\left[\begin{array}{ccccc} x_{11}^{\prime } & \cdots & x_{1k}^{\prime } & \cdots & x_{1\overset{˘}{u}}^{\prime }\\ \vdots & \ddots & \vdots & \ddots & \vdots \\ x_{i1}^{\prime } & \cdots & x_{ik}^{\prime } & \cdots & x_{i\overset{˘}{u}}^{\prime }\\ \vdots & \ddots & \vdots & \ddots & \vdots \\ x_{n1}^{\prime } & \cdots & x_{nk}^{\prime } & \cdots & x_{n\overset{˘}{u}}^{\prime } \end{array}\right]$$

where $X_{i}^{\prime }$ are the latent features for the *i*th business resource, $x_{ik}^{\prime }$ represents the *k*th latent feature of $X_{i}^{\prime }$, and $\overset{˘}{u}$ implies the number of latent features where $\overset{˘}{u}\ll p$.

Based on the above, we use cosine similarity to calculate the similarity between the *i*th and *j*th business resources in $X^{\prime }$. Cosine similarity measures the angle between the corresponding vectors of the *i*th and *j*th business resources in the vector space. The value of cosine similarity ranges from −1 to 1, where a value of 1 signifies complete similarity, 0 indicates no similarity, and −1 denotes complete opposition. The similarity is calculated as follows:

(9) $$\mathrm{sim}{\left(i,j\right)}^{SMBRA}=\frac{\sum_{k=1}^{\overset{˘}{u}}x_{ik}^{\prime }\times x_{jk}^{\prime }}{\sqrt{\sum_{k=1}^{\overset{˘}{u}}{\left(x_{ik}^{\prime }\right)}^{2}}\sqrt{\sum_{k=1}^{\overset{˘}{u}}{\left(x_{jk}^{\prime }\right)}^{2}}}$$

where $\mathrm{sim}{\left(i,j\right)}^{SMBRA}$ is the similarity between the *i*th business resource and the *j*th business resource.

#### 2.1.3. Hybrid Difference-Based Similarity Measure

To improve the efficiency of the similarity measurement in *B*, we use the hybrid difference-based similarity measure (HDSM) proposed by reference [14]. Suppose $B=\left\{Y_{1},Y_{2},\cdots ,Y_{i},\right.$$\left.\cdots ,Y_{j},\cdots ,Y_{n}\right\}$, where $Y_{i}=\left(y_{i1}^{\prime },y_{i2}^{\prime },\ldots ,y_{iu}^{\prime }\right)$ and $Y_{j}=\left(y_{j1}^{\prime },y_{j2}^{\prime },\ldots ,y_{ju}^{\prime }\right)$, respectively, in which some $y_{ik}^{\prime }$ may be missing. The HDSM can be formulated as follows:

(10) $$\mathrm{sim}{\left(i,j\right)}^{HDSM}=1-\frac{R_{i}R_{j}+1}{G}$$

where $R_{i}\left(R_{j}\right)$ is the sum of the non-missing values $y_{ik}^{\prime }\left(y_{jk}^{\prime }\right)$ of $Y_{i}\left(Y_{j}\right)$, and the corresponding $y_{jk}^{\prime }\left(y_{ik}^{\prime }\right)$ represent the missing values. *G* is the product of the two sums of the non-missing values for both $Y_{i}$ and $Y_{j}$:

(11) $$R_{i}=\sum_{\begin{array}{c} y_{ik}^{\prime }\;\mathrm{non}-\mathrm{missing}\;\\ y_{jk}^{\prime }\;\mathrm{missing}\; \end{array}}y_{ik}^{\prime }=\sum_{k\in I_{i}\setminus I_{j}}y_{ik}^{\prime }$$

(12) $$R_{j}=\sum_{\begin{array}{c} y_{jk}^{\prime }\;\mathrm{non}-\mathrm{missing}\;\\ y_{ik}^{\prime }\;\mathrm{missing}\; \end{array}}y_{jk}^{\prime }=\sum_{k\in I_{j}\setminus I_{i}}y_{jk}^{\prime }$$

(13) $$\begin{array}{ccc} G & =\left(\sum_{y_{ik}^{\prime }\;\mathrm{non}-\mathrm{missing}\;}y_{ik}^{\prime }\right)\left(\sum_{y_{jk}^{\prime }\mathrm{non}-\mathrm{missing}}y_{jk}^{\prime }\right)\; & =\left(\sum_{k\in I_{i}}y_{ik}^{\prime }\right)\left(\sum_{k\in I_{j}}y_{jk}^{\prime }\right) \end{array}$$

where $I_{i}\left(I_{j}\right)$ denotes the set of (non-missing) values for the *i*th business resource (the *j*th business resource), and ‘∖’ is the complement operator in the set theory.

#### 2.1.4. Comprehensive Similarity and the Predicted Value

When there are more missing values, we use the HDSM to calculate similarity; when there are fewer missing values, we integrate the SMBRA and the HDSM, to accurately calculate the similarity between business resources. In the case of a few missing values, the algorithm should smoothly transition to using the original data values for the similarity calculation. The sigmoid function is used to conduct the smoothing process, in order to calculate the final similarity. The final similarity calculation is as follows:

(14) $$FS_{ij}=\sigma \cdot \mathrm{sim}{\left(i,j\right)}^{SMBRA}+\left(1-\sigma \right)\cdot \mathrm{sim}{\left(i,j\right)}^{HDSM}$$

(15) $$\sigma =2\times \left(1-\frac{1}{1+\exp\left(-\left|I_{i}\right|\right)}\right)$$

where σ is the sigmoid function.

The final similarity is utilized to establish the nearest neighbor set of the target business resource by selecting the business resources with the highest similarity values. These nearest neighbors are used to predict the value of item *k* for the *i*th business resource by Equation (16):

(16) $$IR_{ik}=\frac{\sum_{j\in N_{i}}FS_{ij}\times y_{ik}^{\prime }}{\sum_{j\in N_{i}}FS_{ij}}$$

where $N_{i}$ is the nearest neighbors set of the *i*th business resource and $IR_{ik}$ denotes the predicted value of the *i*th business resource on item *k*. The predicted value $IR_{ik}$ is used to fill in the missing value for the *i*th business resource on item *k*.

### 2.2. Constructing a Satisfaction Evaluation Index System

The evaluation indices of satisfaction are important for BMBR. We carefully analyze the demander’s requirements and the supplier’s preferences, and we then construct a satisfaction evaluation index system.

#### 2.2.1. Matching Analysis Based on Business Resource Demanders

Business resource demander-based matching aims to find the optimal business resources that meet the business requirements of the demander from a huge amount of information resources [2]. On the third-party cloud platform, it is influenced by several factors [19]. These factors include the quality, price, and timeliness of the resources, as well as the service capability, fulfillment capability, and responsiveness of the supplier. In summary, the satisfaction evaluation indices of the demander are shown in Table 1.

In Table 1, $rv_{1},rv_{2},rv_{3},rv_{4},rv_{5}$, and $rv_{6}$ indicate the evaluation index values. They are as follows:

(17) $$rv_{1}=1-\frac{N_{r}}{N_{s}}$$

(18) $$rv_{2}=P_{r}+C_{c}$$

(19) $$rv_{3}=T_{r}+T_{t}$$

(20) $$rv_{4}=S_{o}+\left(1-S_{c}\right)$$

In Equation (17), $rv_{1}$ represents the value of $ra_{1},N_{s}$ is the total sales volume of the business resources, and $N_{r}$ is the return quantity. In Equation (18), $rv_{2}$ denotes the value of $ra_{2},P_{r}$ is the price of the business resources, and $C_{c}$ is the collaboration cost. In Equation (19), $rv_{3}$ denotes the value of $ra_{3},T_{r}$ is the response time, and $T_{t}$ is the delivery time. In Equation (20), $rv_{4}$ denotes the value of $ra_{4},S_{o}$ indicates an on-time delivery rate for the business resources, $S_{c}$ indicates the complaint rate of the business resources, $rv_{5}$ denotes the value of $ra_{5}$, and $rv_{6}$ is the value of $ra_{6}$. They are determined qualitatively by the demanders.

#### 2.2.2. Matching Analysis Based on Business Resource Suppliers

Business resource supplier-based matching aims to select the best demander that meets the supplier’s preference from the demanders [2]. The satisfaction evaluation indices of the supplier are shown in Table 2:

In Table 2, $cv_{1}$, $cv_{2}$, and $cv_{3}$ indicate the evaluation index values. They are determined qualitatively by the suppliers.

### 2.3. Normalization of the Satisfaction Evaluation Index Values

In order to eliminate the dimensional and magnitude differences between different indicators, it is necessary to normalize the satisfaction evaluation indicator data [2]. We use the data from *B* to form the quantified satisfaction evaluation matrices RV and CV.RV is a satisfaction evaluation matrix based on business resource suppliers. RV is shown below:

(21) $$RV=\left[\begin{array}{ccccc} rv_{11} & \cdots & rv_{1k} & \cdots & rv_{1u}\\ \vdots & \ddots & \vdots & \ddots & \vdots \\ rv_{i1} & \cdots & rv_{ik} & \cdots & rv_{iu}\\ \vdots & \ddots & \vdots & \ddots & \vdots \\ rv_{n1} & \cdots & rv_{nk} & \cdots & rv_{nu} \end{array}\right]$$

where $rv_{ik}$ represents the *k*th index value of the *i*th business resource in the RV matrix. Similarly, $CV\in R^{m\times n}$ is a satisfaction evaluation matrix based on business resource suppliers. Its elements are represented as $cv_{ik}$, where 1≤i≤n and 1≤k≤v; $cv_{ik}$ is the *k*th index value of the *i*th business resource in the CV matrix. Since the satisfaction evaluation indices all have different scales, it is necessary to normalize all the index values. Additionally, any contrary negative indicator values are converted to positive indicator values, to address the inconsistency of the index types:

(22) $$dv_{ik}^{\prime }=\left\{\begin{array}{cc} \frac{dv_{ik}-dv_{\min}}{dv_{\max}-dv_{\min}}, & dv_{\max}-dv_{\min}\neq 0\\ 1 & ,dv_{\max}-dv_{\min}=0 \end{array}\right.$$

(23) $$dv_{ik}^{\prime }=\left\{\begin{array}{cc} \frac{dv_{\max}-dv_{ik}}{dv_{\max}-dv_{\min}}, & dv_{\max}-dv_{\min}\neq 0\\ 1 & ,dv_{\max}-dv_{\min}=0 \end{array}\right.$$

where $dv_{ik}$ is $rv_{ik}$ or $cv_{ik},dv_{ik}^{\prime }$ is the normalized value of $dv_{ik}$, which falls within the range [0, 1]; $dv_{\max}$ is the maximum value in the set $\left\{dv_{1k},\ldots ,dv_{ik},\ldots ,dv_{nk}\right\}$; and $dv_{\min}$ is the minimum value in the set $\left\{dv_{1k},\ldots ,dv_{ik},\ldots ,dv_{nk}\right\}$.

RV and CV are converted to the corresponding normalized satisfaction evaluation matrices $RV^{\prime }$ and $CV^{\prime }$ by Equations (22) and (23). $RV^{\prime }$ and $CV^{\prime }$ inherit the same dimensions, and their elements are denoted as $rv_{ik}^{\prime }$ and $cv_{ik}^{\prime }$.

### 2.4. Determination of Weights

The determination of weights is a crucial step in bilateral matching. In business co-operation, the demands and suppliers of business resources have different preferences for the satisfaction evaluation indices, due to different perspectives of consideration. We use the FAHP to calculate the subjective weights of the evaluation indicators, in order to reflect the demands and preferences of both the demanders and the suppliers. However, using only subjective weights cannot reflect the objective differences in the evaluation indices. In order to more fully reflect the rationality of weighting, we use the EWM to calculate the objective weight of the evaluation indices.

#### 2.4.1. FAHP

The FAHP is a decision analysis method that combines fuzzy theory and the analytic hierarchy process (AHP), which is mainly used to deal with complex decision factors with ambiguity and uncertainty. It is mainly used to evaluate the weight of multi-factor influences, especially when there are subjective judgments and ambiguities between factors. It determines the subjective weight through the following steps:

- Build Fuzzy Complementary Judgment Matrix

Assume that there is a set of relevant factors in the evaluation indicators ${ra}_{k}$(k=1,2,…,u). Using the 0.1–0.9 scaling method shown in Table 3 for quantitative scaling, the fuzzy complementary judgment matrix $FI={\left[fi_{kk^{\prime }}\right]}_{u\times u}$ is obtained. The element of the FI denotes the importance of $ra_{k}$ compared with $ra_{k^{\prime }}$, $0\leq fi_{kk^{\prime }}\leq 1$ ( $1\leq k\leq k^{\prime }\leq u$), $fi_{kk^{\prime }}+fi_{k^{\prime }k}=1$, $fi_{kk}=0.5$. The FI serves as a crucial instrument for assessing the importance of the factor set. It is constructed by integrating the evaluation results of experts on the significance of each factor within the set of evaluation index factors.

- Weight Calculation

According to the opinions of *k* different experts, $n^{\prime }$ different fuzzy judgment matrices are constructed, denoted as $FI_{1},FI_{2},\ldots ,FI_{l^{\prime }},\ldots ,FI_{n^{\prime }}$. The weight of the *k*th metric provided by the $l^{\prime }$th expert opinion is calculated according to Equation (24):

(24) $$w_{k}=\frac{\sum_{k^{\prime }=1}^{u}{fi}_{kk^{\prime }}+\frac{u}{2}-1}{u(u-1)}$$

- Consistency Test

In order to determine whether the weights calculated according to Equation (24) are reasonable, it is necessary to perform a consistency test. The compatibility index between the judgment matrix and the weight matrix is as follows:

(25) $$I(FI,W^{*})=\frac{1}{u^{2}}\sum_{k=1}^{u}\sum_{k^{\prime }=1}^{u}\left|fi_{kk^{\prime }}+w_{k^{\prime }k}-1\right|$$

(26) $$W^{*}={\left(w_{kk^{\prime }}\right)}_{u\times u}$$

(27) $$w_{kk^{\prime }}=w_{k}/\left(w_{k}+w_{k^{\prime }}\right)$$

If the value of the compatibility index is less than 0.1, the judgment matrix could be considered to have satisfactory consistency.

- Subjective Empowerment

The subjective weight vector $W^{s_{1}}=\left(w_{1}^{s_{1}},w_{2}^{s_{1}},\cdots ,w_{u}^{s_{1}}\right)$ is obtained by combining the opinions of all experts through the maximum characteristic root method. The detailed steps of the maximum characteristic root method are described in the previous literature [20]. Similarly, the subjective weight vector $W^{s_{2}}=\left(w_{1}^{s_{2}},w_{2}^{s_{2}},\cdots ,w_{v}^{s_{2}}\right)$ of the satisfaction evaluation indices based on the business resource suppliers is obtained.

#### 2.4.2. EWM

The EWM is one of the classic algorithms for calculating the weight of the indicator [21]. It determines the weight by calculating the information entropy values of each indicator. For an indicator, the bigger the entropy value is, the smaller the degree of discreteness of the indicator is, the smaller the impact of the indicator [22]. The information entropy is calculated by its definition, as follows:

(28) $$E_{k}=-\frac{1}{\ln n}\cdot \sum_{i=1}^{n}p_{ik}\cdot \log p_{ik}$$

where $p_{ik}$ represents the index of the *i*th business resource under the *k*th indicator. And its formula is defined as follows:

(29) $$p_{ik}=\frac{rv_{ik}^{\prime }}{\sum_{i=1}^{n}{rv}_{ik}^{\prime }}$$

The weight of the *k*th satisfaction evaluation index based on the business resource demanders is as follows:

(30) $$w_{k}^{d_{1}}=\frac{1-E_{k}}{\sum_{k=1}^{m}\left(1-E_{k}\right)}$$

Similarly, the weight $w_{k}^{s_{1}}$ of the *k*th evaluation indicators based on the suppliers is obtained.

### 2.5. Construction and Solution of a Multi-Objective Optimization Model

The purpose of the bilateral matching for business resources is to ensure that both the demander and the provider participants achieve maximum satisfaction. Given the collaboration requirements between the demander and the provider, this paper integrates the synergy effect into a two-sided matching strategy.

#### 2.5.1. Construction of Multi-Objective Optimization Model

Based on the $RV^{\prime }$ and $CV^{\prime }$, the supplier’s maximum matching satisfaction and the demander’s maximum matching satisfaction are as follows:

(31) $$\max SF_{1}\left(sr_{i}\right)=1-\sqrt{\sum_{k=1}^{u}{\left(w_{k}^{d_{1}}\times rv_{k}^{{*}^{\prime }}-w_{k}^{s_{1}}\times rv_{ik}^{\prime }\right)}^{2}}$$

(32) $$\max SF_{2}\left(sr_{i}\right)=1-\sqrt{\sum_{l=1}^{v}{\left(w_{l}^{s_{2}}\times cv_{il}^{\prime }-w_{l}^{d_{2}}\times cv_{l}^{{*}^{\prime }}\right)}^{2}}$$

$$\begin{array}{c} \;s.t.\;w_{k}^{d_{1}}\geq 0,w_{k}^{s_{1}}\geq 0,\sum_{k=1}^{u}w_{k}^{d_{1}}=1,\sum_{k=1}^{u}w_{k}^{s_{1}}=1,\\ w_{l}^{s_{2}}\geq 0,w_{l}^{d_{2}}\geq 0,\sum_{l=1}^{v}w_{l}^{s_{2}}=1,\sum_{l=1}^{v}w_{l}^{d_{2}}=1 \end{array}$$

where $sr_{i}$ is the *i*th business resource; $\max SF_{1}\left(sr_{i}\right)$ represents the maximization of demander satisfaction in business resource matching; $\max SF_{2}\left(sr_{i}\right)$ denotes the maximization of supplier satisfaction in business resource matching; $rv_{k}^{{*}^{\prime }}$ refers to the normalized input value of the *k*th satisfaction evaluation index from the demander of business resources; $w_{k}^{d_{1}}$ is the subjective weight of $rv_{k}^{{*}^{\prime }};w_{k}^{s_{1}}$ is the objective weight of $rv_{ik}^{\prime }$; $cv_{l}^{{*}^{\prime }}$ is the normalized input value of the *k*th satisfaction evaluation index from the supplier of business resources; $w_{l}^{s_{2}}$ is the objective weight of $cv_{il}^{\prime }$; $w_{l}^{d_{2}}$ is the subjective weight of $cv_{l}^{{*}^{\prime }}$. We utilize the above FAHP to determine the subjective weight. Additionally, we employ the above EWM to calculate the objective weight.

In order to help enterprises accurately obtain high-quality and valuable business resources on the third-party cloud platform, it is necessary to consider the collaboration requirements between business resource demanders and suppliers [12,23]. The better the synergy between business resource demander *u* and supplier *v*, the higher the likelihood of *u* choosing the *i*th business resource provided by *v*. Therefore, in this study, the synergy satisfaction of *u* choosing *i* is measured by the degree of synergy effect between *u* and *v*. The synergy effect is mainly expressed in three aspects: co-community relationship, transaction performance, and business resource-sharing ability.

Communities include various cloud platforms, alliances, and online groups [24]. The strength of the common community relationship QS depends on the number of common communities in which both *u* and *v* participate:

(33) $$QS=\frac{\left|NR_{u}\cap NR_{v}\right|}{\left|NR_{u}\cup NR_{v}\right|}$$

where $NS_{u}\left(NR_{v}\right)$ is a set of communities in which u(v) participates.

Transaction performance can reduce transaction costs and increase service efficiency [25]. The interactive transaction strength CS is dependent on the total transaction volume TA in all periods and the cooperation activities CA in the current period. CS can be calculated as follows:

(34) $$CS=\eta_{1}\frac{\sum_{\overset{˘}{k}=1}^{m}CT_{uv}^{\overset{˘}{k}}\times N_{uv}^{\overset{˘}{k}}}{\max_{f,v}\sum_{\overset{˘}{k}=1}^{m}CT_{fv}^{\overset{˘}{k}}\times N_{fv}^{\overset{˘}{k}}}+\eta_{2}\frac{CT_{uv}^{\mathrm{now}\;}}{\max_{f,v}^{m}\sum_{\overset{˘}{k}=1}^{m}CT_{uv}^{\mathrm{now}}},f=1,2,\ldots ,n$$

where $CT_{uv}^{\overset{˘}{k}}$ and $N_{uv}^{\overset{˘}{k}}$ are the number and the single transaction volume of the transactions in the $\overset{˘}{k}$th period, respectively; *f* denotes the supplier corresponding to the business resource; $CT_{uv}^{\mathrm{now}}$ is the transaction volume between *u* and *v* in the current period; and $\eta_{1}$ and $\eta_{2}$ indicate the relevant criteria weights.

Business resource sharing ability means the level of information sharing. It is shown as follows:

(35) $$IS=\frac{Nt_{uv}}{Pt}$$

where IS denotes business resource sharing capacity; $Nt_{uv}$ represents the number of times *u* uses the business resources provided by *v*. Pt is a fixed period.

In conclusion, the synergy satisfaction is shown below:

(36) $$SF_{3}\left(sr_{i}\right)=\delta_{1}QS_{uv}^{\prime }+\delta_{2}CS_{uv}^{\prime }+\delta_{3}IS_{u}^{\prime }$$

where $QS_{uv}^{\prime },CS_{uv}^{\prime },IS_{u}^{\prime }$ are normalized numbers using Equation (22), and $\delta_{1},\delta_{2},\delta_{3}$ denote the weights of $QS_{uv}^{\prime },CS_{uv}^{\prime },IS_{u}^{\prime }$ respectively.

#### 2.5.2. Solution of Multi-Objective Matching Model

For resolving this multi-objective optimization model, the linear weighting method is exploited, to convert the multi-objective model into a single-objective optimization model [25]. This is shown as Equation (37):

(37) $$\begin{array}{cc} \max SF(sr_{i}) & =\theta_{1}\times SF_{1}\left(sr_{i}\right)+\theta_{2}\times SF_{2}\left(sr_{i}\right)+\theta_{3}\times SF_{3}\left(sr_{i}\right)\\ s.t.\; & \theta_{1}\geq 0,\theta_{2}\geq 0,\theta_{3}\geq 0,\\ & \theta_{1}+\theta_{2}+\theta_{3}=1 \end{array}$$

where $\theta_{1}$, $\theta_{2}$, $\theta_{3}$ are the weights of $SF_{1}\left(sr_{i}\right)$, $SF_{2}\left(sr_{i}\right)$, $SF_{3}\left(sr_{i}\right)$, respectively. By default, $\theta_{1}$ = $\theta_{2}$ = $\theta_{3}$ = 1/3. However, in the actual business environment, the values of $\theta_{1}$, $\theta_{2}$, and $\theta_{3}$ can be determined based on the specific requirements of both the demander and the supplier.

## 3. Example Application and Comparison Analysis

For this section, an example of bilateral matching on the “ASP/SaaS-based manufacturing value chain collaboration platform” was applied, to verify the effectiveness of the proposed method. Furthermore, to evaluate the performance, we compared it with other state-of-the-art matching methods.

To implement the proposed method, we utilized the Python 3.9 programming language in Anaconda software version 2021. We obtained 300,000 customer transaction data of parts agents from the ASP/SaaS-based manufacturing industry value chain collaboration platform for 2019–2021 [2,26]. We extracted data resources related to auto parts from the data space of the platform, to form six datasets [2]. The first dataset, consisting of “Engine Parts”, was called dataset_1. The second dataset, consisting of “Clutch and Transmission Parts”, was called dataset_2. The third dataset, consisting of “Hydraulic Lift Parts”, was called dataset_3. The fourth dataset, consisting of “Body and Interior/Exterior Parts”, was called dataset_4. The fifth dataset, consisting of “Electrical Parts”, was called dataset_5. The sixth dataset, consisting of “Brake Parts”, was called dataset_6. We set the following core parameters for the TextCNN: the convolution sizes were 2, 3, and 4; the number of filters was 100; the dropout rate was 0.5; and the batch size was 128. The feature dimension of the word was 100, the window size was 5, and the minimum word frequency for truncation was 5.

### 3.1. Example Application

The paper employed the business resources of engine parts as a case study to validate the proposed method. Specifically, we randomly selected 12,000 data resources related to auto parts from dataset_1, which constituted matrix *B*, as presented in Table 4. To process the textual data in Table 4, we utilized the textCNN for quantifying the text information. Subsequently, we presented the resulting quantized data in the same table. For Table 4, we applied the CSKI approach to addressing missing values, which led to the generation of an updated Table 4 displaying the results obtained after filling in the missing values.

The paper took the business data resource requirements of an automobile after-sales service enterprise $ds_{1}$ for a certain engine part as an example, to verify the feasibility and effectiveness of the proposed method. Through preliminary screening, eight business resources from different suppliers were identified: namely, $rs_{1}$, $rs_{2}$, $rs_{3}$, $rs_{4}$, $rs_{5}$, $rs_{6}$, $rs_{7}$, $rs_{8}$. Subsequently, bilateral matching of business resources was realized. The data from Table 4 was utilized to construct satisfaction evaluation matrices RV and CV in Table 5, based on the satisfaction evaluation index system. Subsequently, normalized satisfaction matrices, denoted as $RV^{\prime }$ and $CV^{\prime }$, were derived from Table 5, using Equations (22) and (23). These normalized matrices are presented in Table 6.

The data from Table 4 were used in Equations (33)–(35) to calculate $QS^{\prime },CS^{\prime },IS^{\prime }$. The weight coefficients were set as $\delta_{1}=\delta_{2}=\delta_{3}=1/3$ for solving the mathematical optimization model [25]. These weights and data were then applied in Equation (36) to calculate $SF_{3}$. The corresponding results are presented in Table 7.

**Figure 2:**
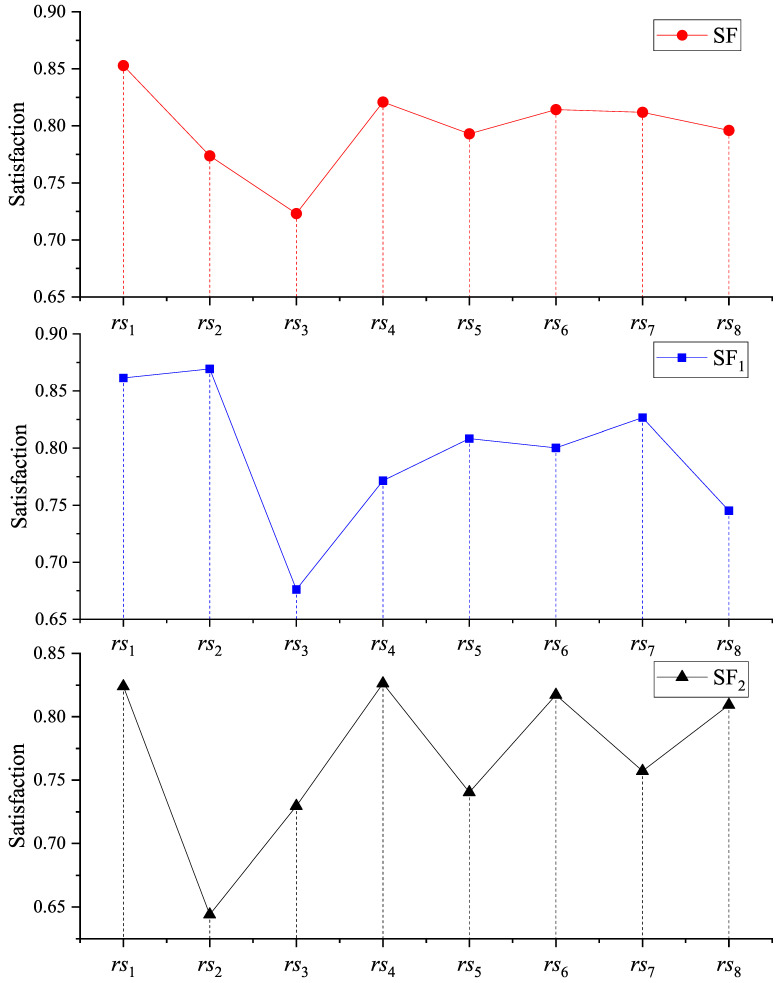
The results of $SF_{1}$, $SF_{2}$, and SF.

The subjective weights of the evaluation indices $ra_{1}-ra_{6}$ were calculated by the FAHP. They were $w_{1}^{d_{1}}=0.1911,w_{2}^{d_{1}}=0.1667,w_{3}^{d_{1}}=0.1581,w_{4}^{d_{1}}=0.1734,w_{5}^{d_{1}}=0.1644$, and $w_{6}^{d_{1}}=0.1463$. The objective weights of the evaluation indices $ra_{1}-ra_{6}$ were calculated by the EWM. They were $w_{1}^{s_{1}}=0.1011,w_{2}^{s_{1}}=0.1312,w_{3}^{s_{1}}=0.1472,w_{4}^{s_{1}}=0.1534,w_{5}^{s_{1}}=0.3249$, and $w_{6}^{s_{1}}=0.1422$. The subjective weights of the evaluation indices $ca_{1}-ca_{3}$ were calculated by the FAHP. They were $w_{1}^{d_{2}}=0.3172,w_{2}^{d_{2}}=0.3538$, and $w_{3}^{d_{2}}=0.329$. The objective weights of the evaluation indices $ca_{1}-ca_{3}$ were calculated by the EWM. They were $w_{1}^{s_{2}}=0.3289,w_{2}^{s_{2}}=0.3356$, and $w_{3}^{s_{2}}=0.3355$. The weights mentioned above, along with the data from Table 6 and Table 7, were used in Equations (31), (32) and (37) to calculate $SF_{1},SF_{2}$, and SF. The detailed results can be found in Figure 2. After performing descending sorting on the satisfaction values corresponding to different dimensions, the sorting results of $SF_{1},SF_{2}$, and SF were obtained. The sorting result of $SF_{1}$ was $rs_{2}$>$rs_{1}$>$rs_{7}$>$rs_{5}$>$rs_{6}$>$rs_{4}$>$rs_{8}$>$rs_{3}$. The sorting result of $SF_{2}$ was $rs_{4}$>$rs_{1}$>$rs_{6}$>$rs_{8}$>$rs_{7}$>$rs_{5}$>$rs_{3}$>$rs_{2}$. The sorting result of SF was $rs_{1}$>$rs_{4}$>$rs_{6}$>$rs_{7}$>$rs_{8}$>$rs_{5}$>$rs_{2}$>$rs_{3}$.

In Figure 2, it can be observed that in the $SF_{1}$ ranking, $rs_{2}$ had the highest satisfaction, indicating that this resource was most suitable for meeting the demander’s requirements. In the $SF_{2}$ ranking, $rs_{4}$ had the highest satisfaction, indicating that this resource best met the supplier’s requirements. According to the SF ranking, it is evident that $rs_{1}$ had the highest satisfaction, ranking second in both $SF_{1}$ and $SF_{2}$. And $rs_{1}$’s collaborative satisfaction value was significantly better than others. This indicates that BMBR not only meets the requirements of the supplier and demander very well, but also takes into account the synergy effects.

### 3.2. Evaluation Indicators and Comparison Analysis

#### 3.2.1. Evaluation Indicators

To validate the effectiveness of BMBR, we evaluated the performance of the method with accuracy (ACC) and the F1 measure. Accuracy is the proportion of the number of samples that the method predicts correctly over the total number of samples. It can be calculated as

(38) $$F_{ACC}=\frac{TP+TN}{TP+TN+FP+FN}$$

where $F_{ACC}$ indicates the ACC value; TP is the number of positive samples judged as positive; TN is the number of negative samples judged as negative; FP is the number of negative samples judged as positive; and FN is the number of positive samples judged as negative [27]. Positive samples are business resources that are actually used by the demander. Negative samples are business resources that are not used by the demander. The larger the ACC value is, the better the performance of the match is.

The $F_{1}$ measure reflects the overall ability of bilateral matching. A higher $F_{1}$ value indicates better quality of matching. $F_{1}$ can be calculated as

(39) $$F_{1}=\frac{2PR}{P+R}=\frac{2TP}{2TP+FP+FN}$$

(40) $$P=\frac{TP}{TP+FP},\;R=\frac{TP}{TP+FN}$$

where *P* represents Precision and *R* represents Recall.

#### 3.2.2. Comparison Analysis

This paper conducted experiments on six datasets, to evaluate the performance of the proposed method compared with other matching methods. The abbreviations and full terms of the other matching methods are detailed in Table 8.

**Figure 3:**
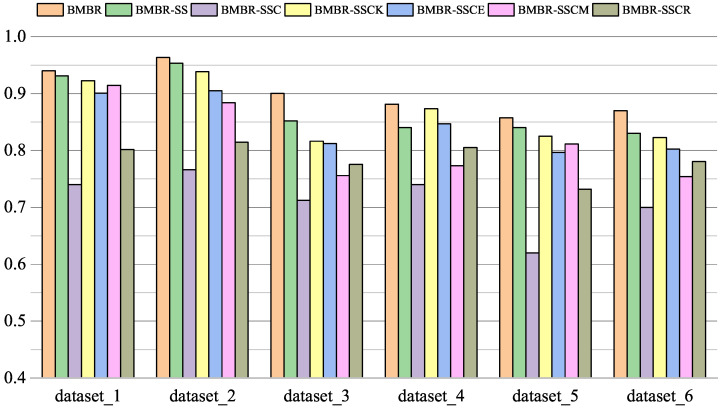
Comparison of different algorithms on different datasets.

To validate the effectiveness of BMBR, we compared BMBR with BMBR-SS, BMBR-SSC, BMBR-SSCK, BMBR-SSCE, BMBR-SSCM, and BMBR-SSCR. The comparison result of the experiment is detailed in Figure 3. As shown in Figure 3, the ACC values of BMBR-SS were higher than BMBR-SSC, BMBR-SSCK, BMBR-SSCE, BMBR-SSCM, and BMBR-SSCR on all six datasets. This indicates that CSKI in BMBR effectively filled in missing values, thereby improving the matching accuracy of BMBR. Additionally, BMBR had a higher ACC value than BMBR-SS, indicating that incorporating synergy effects into the matching method improved the matching performance.

**Figure 4:**
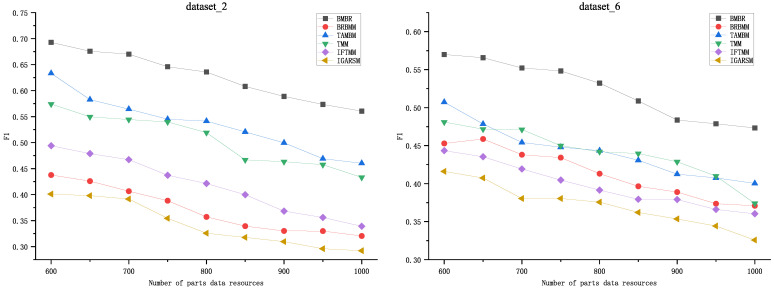
Comparative analysis of business resource matching quality.

To further validate the performance of the proposed method, we compared BMBR with TAMBM, BRBMM, TMM, IGARSM, and IFTMM on two datasets (dataset_2 and dataset_6). As shown in Figure 4, the $F_{1}$ value gradually decreased as the number of business resources increased. And it is easy to see that the $F_{1}$ value of BMBR was significantly higher than that of TAMBM, BRBMM, TMM, and IFTMM on two datasets. This indicates that the matching quality of BMBR is superior to the other five methods. And this indicates that BMBR plays a positive role in improving matching quality.

## 4. Conclusions

On the third-party cloud platform, to help enterprises quickly and accurately obtain high-quality valuable business resources from the complex and massive information resources, we propose a bilateral matching method for business resources based on synergy effects and incomplete data. This method firstly applies CSKI, to address the issue of missing values. Then, it constructs a satisfaction evaluation index system for both supplier and demander, and the weights of the satisfaction evaluation indices are determined based on the FAHP and the EWM. Finally, a bilateral matching model of the business resources is constructed with the objectives of maximizing the matching satisfaction of both the supplier and the demander, as well as achieving the synergy effect. The rationality and effectiveness of the proposed model were validated through experimental analysis, using the engine parts data resource in the automobile after-sales service industry as an example. The superiority effectiveness proposed was verified by comparing with other methods.

Although this research work has some advantages in bilateral matching, there are still some limitations. For example, the proposed method lacks the ability to adjust in real time for dynamic changes of business resources on the third-party cloud platform. In our future work, we will introduce adaptive algorithms, to make the matching method dynamically adaptable, to respond to data changes and user demands in real time. In addition, we will apply this method to other fields, to verify the applicability of the proposed method.

## Figures and Tables

**Table 1:** Satisfaction evaluation index system based on business resources demanders.

Index	Index Value	Index Description	Index Nature
Quality ($ra_{1}$)	$rv_{1}$	The quality of the business resources.	Quantitative Positive
Price ($ra_{2}$)	$rv_{2}$	The price of the business resources.	Quantitative Reverse
Timeliness ($ra_{3}$)	$rv_{3}$	The timeliness with which the suppliers provide the business resources.	Quantitative Reverse
Service capability ($ra_{4}$)	$rv_{4}$	The after-sales service capability of the suppliers.	Quantitative Positive
Fulfillment Capability ($ra_{5}$)	$rv_{5}$	The supplier’s ability to perform the contract.	Qualitative Positive
Responsiveness ($ra_{6}$)	$rv_{6}$	The supplier’s responsiveness to the business resource needs.	Qualitative Positive

**Table 2:** Satisfaction evaluation index system based on business resource suppliers.

Index	Index Value	Index Description	Index Nature
Reputation ($ca_{1}$)	$cv_{1}$	The corporate reputation of the demander.	Qualitative Positive
Payment speed ($ca_{2}$)	$cv_{2}$	The speed at which the demander pays.	Qualitative Positive
Collaboration potential ($ca_{3}$)	$cv_{3}$	The long-term cooperation capacity of the demander.	Qualitative Positive

**Table 3:** 0.1–0.9 scale method and its meaning.

Scale	Meaning of Scale
0.5	$ra_{k}$ and $ra_{k^{\prime }}$ are equally important
0.6	$ra_{k}$ is slightly more important than $ra_{k^{\prime }}$
0.7	$ra_{k}$ is generally more important than $ra_{k^{\prime }}$
0.8	$ra_{k}$ is much more important than $ra_{k^{\prime }}$
0.9	$ra_{k}$ is more important than $ra_{k^{\prime }}$
0.1, 0.2, 0.3, 0.4	y = 1 − x

**Table 4:** Parts business resource.

Business Resource	Return Quantity	Return Quantity (after Filling)	Total Volume sales	Total Volume Sales (after Filling)	…	Response Speed	Response Speed (Quantified Value)	…
$rs_{1}$	599	599		19,484	…	Responded more promptly…	5	…
$rs_{2}$	192	192	15,500	15,500	…	The company responded quickly …	4	…
…	…	…	…	…	…	…	…	…
$rs_{1199}$	525	525	10,014	10,014	…	Had a faster response time …	4	…
$rs_{1200}$		328	16,561	16,561	…	The response speed was general…	3	…

**Table 5:** Satisfaction matrices of business resources.

	*RV*							*CV*		
**Business resource**	$rv_{1}$	$rv_{2}$	$rv_{3}$	$rv_{4}$	$rv_{5}$	$rv_{6}$		$cv_{1}$	$cv_{2}$	$cv_{3}$
$rs_{1}$	0.9952	37.68	10.7	1.7	2	4		4	4	5
$rs_{2}$	0.9847	34.2	2.4	1.71	3	4		3	1	4
$rs_{3}$	0.9111	33.6	31.5	1.6	1	1		1	4	2
$rs_{4}$	0.9575	45.88	9.4	1.71	2	2		5	3	3
$rs_{5}$	0.9910	39.4	23.4	1.79	1	3		4	2	4
$rs_{6}$	0.9886	40.61	4.5	1.64	2	3		5	5	1
$rs_{7}$	0.9834	39.6	20.7	1.71	2	3		2	4	5
$rs_{8}$	0.9916	39.83	17.4	1.72	5	3		5	3	4
**Matching request**	$rv_{1}^{*}$	$rv_{2}^{*}$	$rv_{3}^{*}$	$rv_{4}^{*}$	$rv_{5}^{*}$	$rv_{6}^{*}$		$cv_{1}^{*}$	$cv_{2}^{*}$	$cv_{3}^{*}$
$ds_{1}$	0.98	35	10	1.8	4	5		4	5	3

**Table 6:** Normalized satisfaction matrices of business resources.

	*RV*							*CV*		
**Business resource**	$rv_{1}^{\prime }$	$rv_{2}^{\prime }$	$rv_{3}^{\prime }$	$rv_{4}^{\prime }$	$rv_{5}^{\prime }$	$rv_{6}^{\prime }$		$cv_{1}^{\prime }$	$cv_{2}^{\prime }$	$cv_{3}^{\prime }$
$rs_{1}$	1	0.6393	0.7148	0.5	0.25	0.75		0.75	0.75	1
$rs_{2}$	0.8751	0.9111	1	0.55	0.5	0.75		0.5	0	0.75
$rs_{3}$	0	1	0	0	0	0		0	0.75	0.25
$rs_{4}$	0.5517	0	0.7595	0.55	0.25	0.25		1	0.5	0.5
$rs_{5}$	0.9501	0.5055	0.2784	0.95	0	0.5		0.75	0.25	0.75
$rs_{6}$	0.9215	0.4111	0.9278	0.2	0.25	0.5		1	1	0
$rs_{7}$	0.8597	0.4899	0.3711	0.55	0.25	0.5		0.25	0.75	1
$rs_{8}$	0.9572	0.4719	0.4845	0.6	1	0.5		1	0.5	0.75
**Matching request**	$rv_{1}^{{*}^{\prime }}$	$rv_{2}^{{*}^{\prime }}$	$rv_{3}^{{*}^{\prime }}$	$rv_{4}^{{*}^{\prime }}$	$rv_{5}^{{*}^{\prime }}$	$rv_{6}^{{*}^{\prime }}$		$cv_{1}^{{*}^{\prime }}$	$cv_{2}^{{*}^{\prime }}$	$cv_{3}^{{*}^{\prime }}$
$ds_{1}$	0.8193	0.8487	0.7388	1	0.75	1		0.75	1	0.5

**Table 7:** Matching satisfaction in different dimensions.

Business Resource	${\mathrm{QS}}^{\prime }$	${\mathrm{CS}}^{\prime }$	${\mathrm{IS}}^{\prime }$	${\mathrm{SF}}_{3}$	${\mathrm{SF}}_{1}$	${\mathrm{SF}}_{2}$	SF
$rs_{1}$	0.8649	0.8134	0.9418	0.8734	0.8614	0.8239	0.8529
$rs_{2}$	0.7268	0.7867	0.9101	0.8079	0.8693	0.6442	0.7738
$rs_{3}$	0.7839	0.6610	0.8466	0.7638	0.6761	0.7296	0.7232
$rs_{4}$	0.7162	0.9256	0.9524	0.8647	0.7716	0.8263	0.8209
$rs_{5}$	0.7379	0.8205	0.9312	0.8299	0.8085	0.7404	0.7929
$rs_{6}$	0.8400	0.7682	0.8677	0.8253	0.8003	0.8171	0.8143
$rs_{7}$	0.7519	0.8571	0.9471	0.8520	0.8267	0.7572	0.8120
$rs_{8}$	0.7745	0.7837	0.9418	0.8333	0.7453	0.8092	0.7959

**Table 8:** Abbreviations and full terms.

Abbreviation	Full Term
TAMBM [5]	task assignment method based on bilateral matching
BRBMM [2]	business resource bilateral matching model
TMM [10]	two-sided matching model
IGARSM [28]	improved genetic algorithm for resource service matching
IFTMM [11]	intuitionistic fuzzy two-sided matching model
BMBR-SS	BMBR without synergy satisfaction
BMBR-SSC	BMBR without synergy satisfaction and CSKI
BMBR-SSCK	BMBR-SSC with k-nearest neighbor
BMBR-SSCE	BMBR-SSC with expectation maximization
BMBR-SSCM	BMBR-SSC with multiple imputation
BMBR-SSCR	BMBR-SSC with regression model

## Data Availability

The data presented in this study are available upon request from the corresponding author. The data are not publicly available, due to copyright.
